# Flavonoid Apigenin Inhibits Lipopolysaccharide-Induced Inflammatory Response through Multiple Mechanisms in Macrophages

**DOI:** 10.1371/journal.pone.0107072

**Published:** 2014-09-05

**Authors:** Xiaoxuan Zhang, Guangji Wang, Emily C. Gurley, Huiping Zhou

**Affiliations:** 1 Center of Drug Metabolism and Pharmacokinetics, China Pharmaceutical University, Nanjing, P. R. China; 2 Department of Microbiology & Immunology, Virginia Commonwealth University, Richmond, Virginia, United States of America; 3 Department of Internal Medicine/Gastroenterology and McGuire Veterans Affairs Medical Center, Richmond, Virginia, United States of America; 4 School of Pharmacy, Wenzhou Medical University, Wenzhou, P. R. China; Boston University School of Medicine, United States of America

## Abstract

**Background:**

Apigenin is a non-toxic natural flavonoid that is abundantly present in common fruits and vegetables. It has been reported that apigenin has various beneficial health effects such as anti-inflammation and chemoprevention. Multiple studies have shown that inflammation is an important risk factor for atherosclerosis, diabetes, sepsis, various liver diseases, and other metabolic diseases. Although it has been long realized that apigenin has anti-inflammatory activities, the underlying functional mechanisms are still not fully understood.

**Methodology and Principal Findings:**

In the present study, we examined the effect of apigenin on LPS-induced inflammatory response and further elucidated the potential underlying mechanisms in human THP-1-induced macrophages and mouse J774A.1 macrophages. By using the PrimePCR array, we were able to identify the major target genes regulated by apigenin in LPS-mediated immune response. The results indicated that apigenin significantly inhibited LPS-induced production of pro-inflammatory cytokines, such as IL-6, IL-1β, and TNF-α through modulating multiple intracellular signaling pathways in macrophages. Apigenin inhibited LPS-induced IL-1β production by inhibiting caspase-1 activation through the disruption of the NLRP3 inflammasome assembly. Apigenin also prevented LPS-induced IL-6 and IL-1β production by reducing the mRNA stability *via* inhibiting ERK1/2 activation. In addition, apigenin significantly inhibited TNF-α and IL-1β-induced activation of NF-κB.

**Conclusion and Significance:**

Apigenin Inhibits LPS-induced Inflammatory Response through multiple mechanisms in macrophages. These results provided important scientific evidences for the potential application of apigenin as a therapeutic agent for inflammatory diseases.

## Introduction

Lipopolysaccharide (LPS), a major component of gram-negative bacteria cell membrane, is a well-characterized inducer of the inflammatory response. The initial acute innate immune response to LPS primes the adaptive immune system against further infection [1]. Macrophages are the major players in both innate and adaptive inflammatory responses. It has been well-recognized that the prolonged activation of the inflammatory response contributes to a wide variety of chronic human diseases such as arteriosclerosis, sepsis, obesity, diabetes, various liver diseases, inflammatory bowel disease, autoimmune diseases, allergy and cancer [2], [3]. Activation of macrophages by LPS leads to the increased secretion of a large set of proinflammatory cytokines, such as tumor necrosis factor (TNF)-alpha, interleukin (IL)-1, IL-6, and macrophage chemoattractant protein-1 (MCP-1). Persistent production of these proinflammatory cytokines can cause severe tissue destruction and eventually organ failure. The traditional steroidal anti-inflammatory drugs (SAIDs) and nonsteroidal anti-inflammatory drugs (NSAIDs) are the commonly used to treat acute inflammatory disorders. However, these conventional drugs have not been successful in treating chronic inflammatory diseases due to severe side effects [4]. The need for the development of new anti-inflammatory drugs with higher potency and lower toxicity is urgent to combat various complex inflammatory diseases.

**Figure 1:**
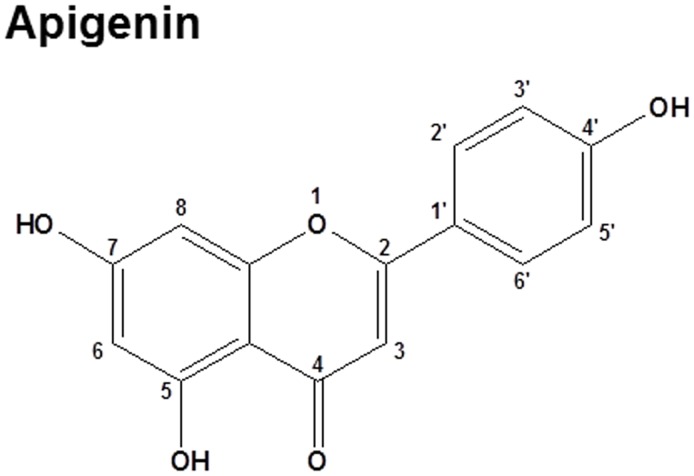
The chemical structure of apigenin.

Flavonoids are a family of polyphenolic compounds, that are widely distributed in the plant kingdom and consumed in significant amounts as part of the human diet [5]. The beneficial effects of these flavonoids to human health have been well-documented [5]–[12]. Epidemiological studies have shown that flavonoids in a healthy diet have potentially beneficial effects in inflammatory diseases and can reduce the risk of various cancers [13]. Apigenin (4′, 5, 7,-trihydroxyflavone) (Fig.1) is a non-toxic and non-mutagenic dietary flavonoid, which is abundantly present in common fruits and vegetables, such as oranges, grapefruits, parsley, onions, chamomile, wheat sprouts, and some seasonings [13], [14]. During last decade, apigenin has garnered increased interest as a health promoting agent because of its low intrinsic toxicity and high chemopreventive efficiency [13], [14]. It has been shown that apigenin induces human pancreatic cancer cell death *via* inhibition of the glycogen synthase kinase-3β/nuclear factor kappa B (NF-κB) signaling pathway [15]. In addition, apigenin has been reported to have anti-inflammatory activities. It protects endothelial cells from LPS-induced inflammation and inhibits allergen-induced airway inflammation [16], [17]. Several intracellular signaling pathways have been suggested to be involved in apigenin-mediated anti-inflammatory effects, such as NF-κB, MAPK/ERK, and JNK pathways [18], [19]. However, the cellular/molecular mechanisms by which apigenin modulates LPS-induced inflammatory response in macrophages have not been fully revealed.

In the present study, we examined the effect of apigenin on LPS-induced inflammatory response in macrophages and further explored the potential cellular/molecular mechanisms involved in its anti-inflammatory effects.

## Methods

### Materials

Apigenin, LPS, phorbol 12-myristate 13-acetate (PMA), actinomycin D, and hygromycin B were purchased from Sigma-Aldrich (St. Louis, MO). Cell Counting Kit-8 (CCK-8) was from Dojindo Molecular Technologies (Kumamoto, Japan). Antibodies against IL-1β, p-ERK1/2, ERK1, ERK2 and ASC were from Santa Cruz Biotechnology (Santa Cruz, CA). Antibody against caspase-1 was from Millipore Corporation (Billerica, MA). Mouse monoclonal antibody against β-actin and NLRP3 polyclonal antibody were from Thermo Scientific (Wilmington, DE). IRDye secondary antibodies were purchased from LI-COR Biosciences (Lincoln, NE). Recombinant human/mouse IL-1β, IL-6 and TNF-α, anti-human/mouse IL-1β, IL-6 and TNF-α antibodies, biotinylated anti-human/mouse IL-1β, IL-6 and TNF-α antibodies, and avidin-HRP were from eBioscience (San Diego, CA). Immune Response Tier 1 H96 Plate, Bio-Rad protein assay reagent, Precision Plus Kaledoscope Standards, iQTM SYBR Green Supermix were obtained from Bio-Rad (Hercules, CA). QIAzol Lysis Reagent was obtained from QIAGEN Sciences (Germantown, MD). High Capacity cDNA Reverse Transcription Kit was from Life Technologies (Grand Island, NY). FuGene HD transfection Reagent, pGL4.32 [luc2P/NF-κB- RE/Hygro] vector and pGL4.76 [hRluc/Hygro] vector were from Promega (Madsion, WI).

### Cell culture and treatment

Human THP-1 monotypic cells (from ATCC, Cat# TIB-202™) were cultured in RPMI 1640 medium supplemented with 10% FBS, 100 U/mLmL penicillin, and 100 µg/mL streptomycin at 37°C with 5% CO2. THP-1 monocytes were treated with PMA (100 ng/mL) for 5 days to induce differentiation into macrophages. Mouse J774A.1 macrophages (from ATCC, Cat# TIB-67™) were maintained in DMEM supplemented with 10% FBS, 100 U/mL penicillin, and 100 µg/mL streptomycin at 37°C with 5% CO2. Cells from passages 12 to 15 were used in these studies. HEK293 cells were cultured in DMEM supplemented with 10% FBS, 0.1 mM non-essential amino acid and 1% Penicillin-Streptomycin. Apigenin was dissolved in dimethyl sulfoxide (DMSO) and directly added into the culture medium. Based on the preliminary toxicity test and previous published studies [20], [21], the concentrations of apigenin used in this study were 6.25, 12.5 and 25 µM. For each result, a minimum of three independent experiments were performed.

### Immune response PrimerPCR assay

Human THP-1-derived macrophages were pretreated with apigenin (25 µM) for 2 h and then treated with LPS (100 ng/mL) for 24 h. Total cellular RNA was isolated using QIAzol Lysis Reagent and reverse transcribed into the first-strand cDNA using a High-Capacity cDNA Transcription Kit as described previously [22]. The 20 ng of cDNA of control or LPS- or LPS + apigenin-treated samples were loaded into the Immune Response Tier 1 H96 plate. The mRNA levels of 91 genes involved in the inflammatory immune response were detected at the same time using iQTM SYBR Green Supermix on Bio-Red CFX96 real-time PCR system. The data were analyzed by using Bio-Rad CFX manager software.

### RNA isolation and quantitative real-time RT-PCR

Cells were pretreated with apigenin for 2 h and then treated with LPS (100 ng/mL) or vehicle control for 24 h. Total Cellular RNA was isolated using QIAzol Lysis Reagent and reverse transcribed into first-strand cDNA using a High-Capacity cDNA Transcription Kit. The mRNA levels of IL-1β, IL-6, TNF-α, caspase-1, ASC, NLRP3 and GAPDH were quantified using gene-specific primers (Table 1). iQTM SYBR Green Supermix was used as a fluorescent dye to detect the presence of double-stranded DNA. The mRNA levels of target genes were quantified with real-time RT-PCR as described previously [22].

**Table 1:** Real-time PCR primers.

Gene	Forward Primer (5′-3′)	Reverse Primer (5′-3′)
hGAPDH	ACATCATCCCTGCCTCTACTGG	TCCGACGCCTGCTTCACC
hIL-1β	TGGCTTATTACAGTGGCAATG	GTGGTGGTCGGAGATTCG
hIL-6	CAGATTTGAGAGTAGTGAGGAAC	CGCAGAATGAGATGAGTTGTC
TNF-α	CGAGTCTGGGCAGGTCTAC	GGGAGGCGTTTGGGAAGG
hCaspase-1	CCACATCCTCAGGCTCAGAAG	GCGGCTTGACTTGTCCATTATTG
hNLRP3	AGGAAGATGATGTTGGACTGG	GTGGATGGGTGGGTTTGG
mGAPDH	GTCGTGGATCTGACGTGCC	GATGCCTGCTTCACCACCTT
mIL-1β	AATCTCACAGCAGCACATC	AGCAGGTTATCATCATCATCC
mIL-6	GAGGATACCACTCCCAACAGACC	AAGTGCATCATCGTTGTTCATACA
mTNF-α	GCCTCCCTCTCATCAGTTC	ACTTGGTGGTTTGCTACG

### Enzyme-Linked Immunosorbent Assay (ELISA)

Cells were pretreated with apigenin for 2 h and then treated with LPS (100 ng/mL) or vehicle control for 24 h. At the end of the treatment, cell culture media were collected and centrifuged at 14,000× rpm for 1 min. The protein levels of TNF-α, IL-1β, and IL-6 in the media were determined by ELISA as described previously [22]. The total protein concentrations of the viable cells were determined using Bio-Rad protein assay reagent. The protein levels of the TNF-α, IL-1β, and IL-6 in media were normalized to the total protein amount of the viable cells and expressed as pg/mg proteins.

### Assessment of IL-1β and IL-6 mRNA stability

Cells were pretreated with apigenin for 2 h and then treated with LPS (100 ng/mL) or vehicle control for 2 h before addition of actinomycin D (5.0 µg/mL, time 0). The cells were harvested for isolation of total cellular RNA at 0.5, 1, 2, 4 and 6 h after addition of actinomycin D. The mRNA stability of IL-1β and IL-6 were quantified with real-time PCR as described previously [23].

### Western blot analysis

Total cell lysates were prepared as previously described [24]. The protein concentration was determined using Bio-Rad protein assay reagent. A total of 70 µg of proteins was resolved on 10% Bis–Tris gels and transferred to nitrocellulose membranes. The protein levels of target genes were detected using specific primary antibodies and IRDye secondary antibodies on Odyssey Fluorescence Imaging System (LI-COR Biosciences, USA). The density of the immunoblots was analyzed using Odyssey V3.0 software and normalized with β-Actin.

### Immunofluorescence staining of ASC

Human THP-1-derived macrophages were cultured on 22×22-mm glass coverslips in 6-well plates. Cells were pretreated with apigenin (25 µM) for 2 h and then treated with LPS (100 ng/ml) or vehicle control for 24 h. Cells were fixed with 3.7% formaldehyde in PBS for 30 min, permeabilized with 0.1% Triton-X-100 for 3 min, and blocked with 3% normal goat serum for 1 h. Rabbit anti-ASC antibody was incubated for 4 h at RT. The negative control was incubated with the same amount of normal rabbit IgG. The Alexa Fluor 488-labeled goat anti-rabbit antibody was incubated for 1 h at RT. The coverslips were mounted with fluorescence mounting medium with DAPI. The intracellular ASC speck formation was visualized under an Olympus 1×71 fluorescence microscope with a 60x oil objective using a dual-filter set for FITC and DAPI. The images were captured using IPLab 4.0 software.

### Measurement of NF-κB activity

NF-κB activity was detected using a NF-κB-luciferase reporter vector, pGL4.32 [luc2P/NF-κB-RE/Hygro]. It contains five copies of a NF-κB response element (NF-κB-RE) that drives the transcription of the luciferase reporter gene luc2P (Photinus pyralis). Briefly, 293 cells were transfected with pGL4.32 [luc2P/NF-κB-RE/Hygro] or pGL4.76 [hRluc/Hygro] using FuGene HD transfection reagent. The stably transfected cells were selected by Hygromycin B (100 µg/mL) for two weeks. To determine the effect of apigenin on inflammatory cytokine-mediated activation of NF-κB, 293 cells expressing NF-κB-RE were pretreated with apigenin for 2 hours, and then treated with human IL-1β (10 ng/mL) or TNF-α (10 ng/mL), or vehicle control for 4 hours. The luciferase activity was detected on a Glomax Multi-functional Plate Reader (Promega) using the Promega Luciferase Assay System kit according to manufacturer's instructions. The relative luciferase activity of each group was compared to control vehicle.

### Statistical analysis

All of the experiments were repeated at least three times and the data were expressed as mean ± SD. One-way ANOVA was employed to analyze the differences between sets of data. To confirm the differences occurred between groups, post hoc tests were used for follow-up test. Statistics were performed using GraphPad Prism 5.0 (GraphPad, San Diego, CA). A value of p<0.05 was considered statistically significant.

## Results

### Identification of the target genes regulated by apigenin in LPS-mediated immune response in macrophages

**Figure 2:**
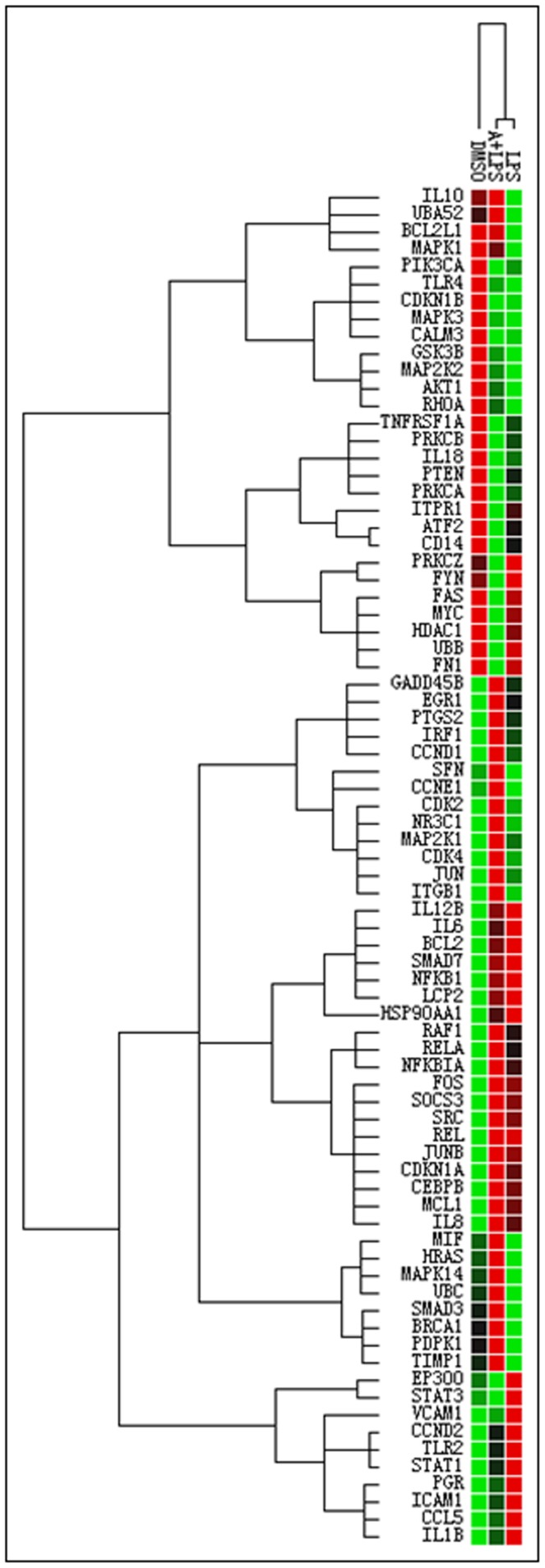
The clustergram of PrimePCR assay in human THP-1-derived macrophages. Cells were pretreated with apigenin (A, 25 µM) or DMSO for 2 h, and then treated with LPS (100 ng/mL) or vehicle control for 24 h. Total cellular RNA was isolated and reverse transcribed. The mRNA levels of 91 genes involved in the inflammatory immune response were detected using a pre-designed PrimePCR assay following the manufacturer's instructions. The results were analyzed using Bio-Rad CFX manager software. The clustergram of all the tested genes is shown.

Although apigenin has been reported to have anti-inflammatory activities and is able to inhibit the expression of several proinflammatory cytokines such as TNF-α and IL-6, there is no information available regarding the effect of apigenin on LPS-induced immune response. In order to determine the effect of apigenin on LPS-induced inflammatory response and identify the specific target genes, we did the quantitative real-time PrimePCR array using the Bio-Rad predesigned assay specifically for inflammatory immune response. The differentiated THP-1 macrophages were pretreated with apigenin (25 µM) for 2 h and then treated with LPS (100 ng/mL) or vehicle control for 24 h. Total cellular RNA was isolated and reverse transcribed into first-strand cDNA. A total of 20 ng of cDNA was used to run a real-time PrimePCR array according to the protocol recommended by the manufacturer. The results indicated that in LPS-stimulated macrophages, more than two dozen genes were significantly up-regulated including IL-6, IL-8, IL1β, IL12β, NFKBIA (nuclear factor of kappa light polypeptide gene enhancer in B-cells inhibitor alpha), and NFKB1. But IL-10 and TLR4 were significantly down-regulated (Fig.2).

**Figure 3:**
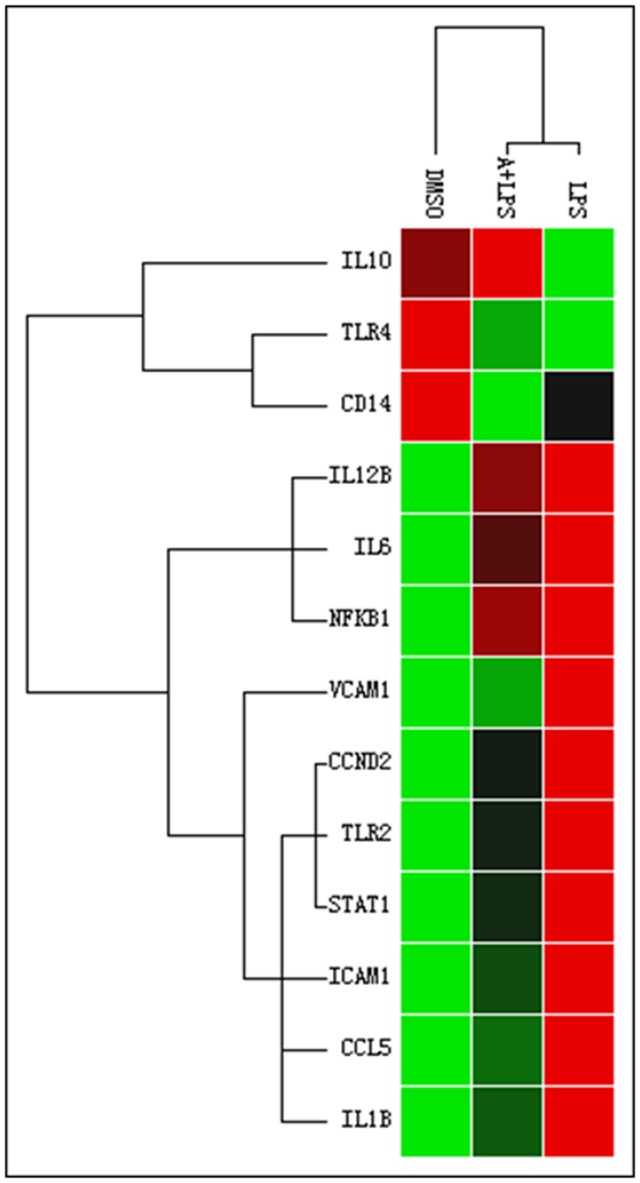
The clustergram of the major target genes of apigenin in human THP-1-derived macrophages. Cells were pretreated with apigenin (A, 25 µM) or DMSO for 2 h, and then treated with LPS (100 ng/mL) or vehicle control for 24 h. Total cellular RNA was isolated and reverse transcribed. The mRNA levels of 91 genes involved in an inflammatory immune response were detected using a pre-designed PrimePCR assay following the manufacturer's instructions. The results were analyzed using Bio-Rad CFX manager software. The clustergram of major target genes is shown.

Cytokines are important immunomodulation agents in regulating host responses to infection, inflammation, sepsis, and cancer [25]. As shown in Fig.3, apigenin not only significantly inhibited LPS-induced up-regulation of pro-inflammatory cytokines, such as IL-1β, IL-6, and IL-12β, but also reduced LPS-induced increase of inflammatory chemokine CCL5 and adhesion molecules (ICAM1 and VCAM1). In addition, LPS-induced down-regulation of IL-10 was reversed by apigenin. These results suggest that apigenin may modulate LPS-induced inflammatory response through multiple mechanisms.

### Effect of apigenin on LPS-induced expression of pro-inflammatory cytokines in macrophages

**Figure 4:**
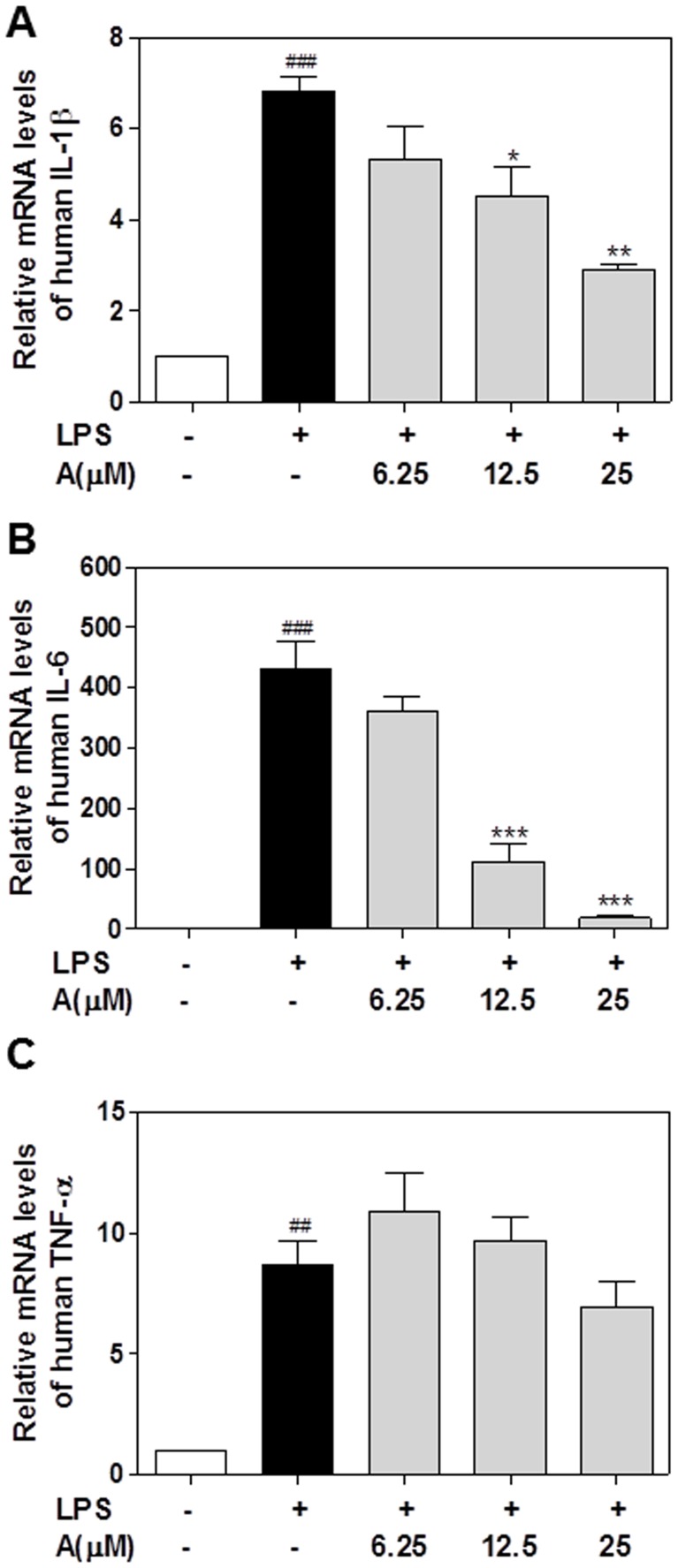
Effect of apigenin on LPS-induced proinflammatory cytokines mRNA expression in human THP-1-derived macrophages. Cells were pretreated with different concentrations of apigenin (A, 6.25, 12.5,25 µM) for 2 h, then treated with LPS (100 ng/mL) for 24 hours. Total cellular RNA was isolated and reverse transcribed. The relative mRNA levels of IL-1β, IL-6, and TNF-α were detected by real-time RT-PCR as described under “Methods”. Values are mean ± S.E. of three independent experiments. Statistical significance relative to vehicle control, ##p<0.01; ###p<0.001; Statistical significance relative to LPS group, *p<0.05, **p<0.01, ***p<0.001. **A**. IL-1β; **B**. IL-6; **C**. TNF-α.

**Figure 5:**
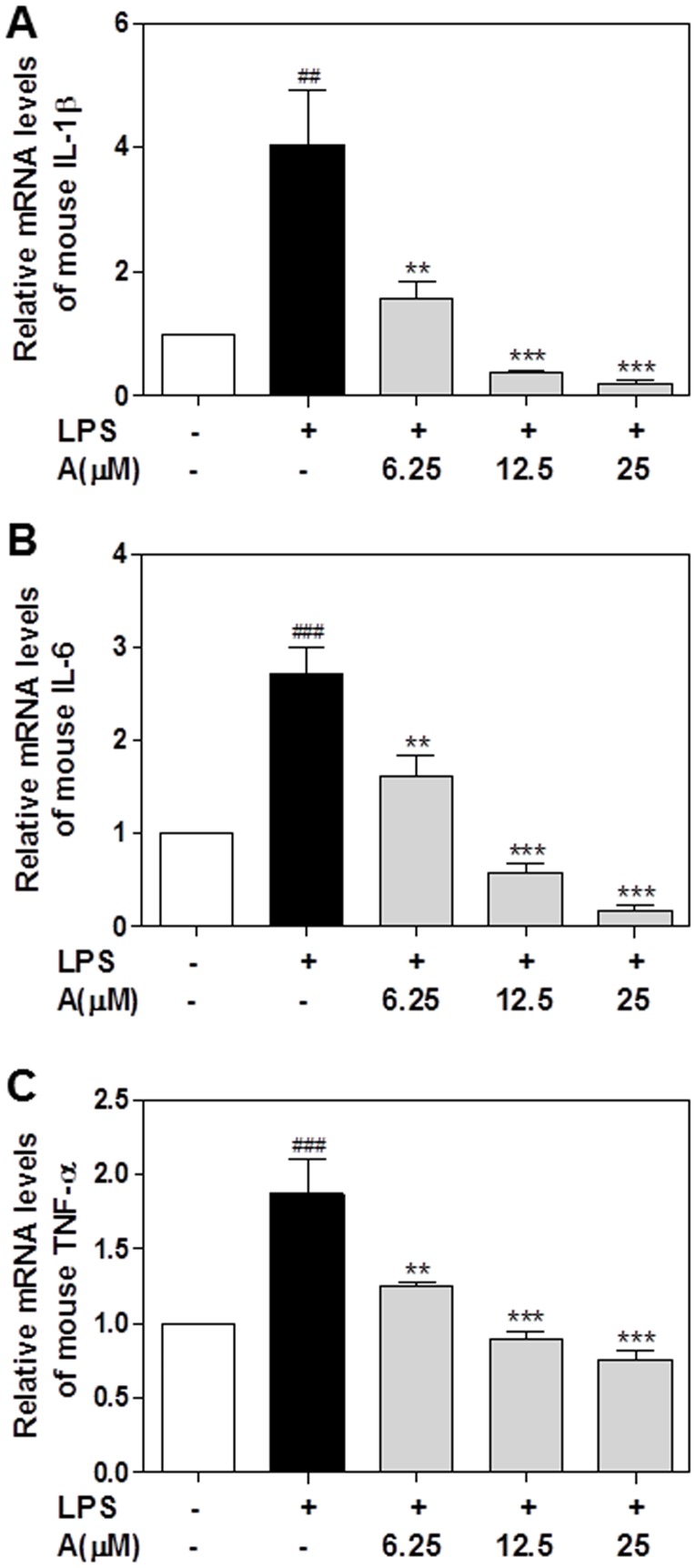
Effect of apigenin on LPS-induced proinflammatory cytokines mRNA expression in mouse J774A.1 macrophages. Cells were pretreated with different concentrations of apigenin (A, 6.25, 12.5,25 µM) for 2 h and then treated with LPS (100 ng/mL) for 24 hours. Total cellular RNA was isolated and reverse transcribed. The relative mRNA levels of IL-1β, IL-6, and TNF-α were detected by real-time RT-PCR as described under “Methods”. Values are mean ± S.E. of three independent experiments. Statistical significance relative to vehicle control, ##p<0.01; ###p<0.001; Statistical significance relative to LPS group, **p<0.01, ***p<0.001. **A**. IL-1β; **B**. IL-6; **C**. TNF-α.

In order to verify the results of PrimePCR Array and confirm the anti-inflammatory effect of apigenin in macrophages, we measured the mRNA levels of several key pro-inflammatory cytokines involved in inflammatory response including IL-1β, TNF-α, and IL-6 using real-time RT-PCR [22], [26], [27]. As shown in Fig.4, LPS markedly increased mRNA levels of IL-1β, IL-6 and TNF-α in human THP-1-derived macrophages, which were completely inhibited by apigenin in a dose-dependent manner. Similarly, apigenin also markedly inhibited LPS-induced expression of IL-1β, IL-6, and TNF-α in a dose-dependent manner in mouse J774A.1 macrophages (Fig.5).

### Effect of apigenin on LPS-induced secretion of IL-6, TNF-α and IL-1β protein in macrophages

**Figure 6:**
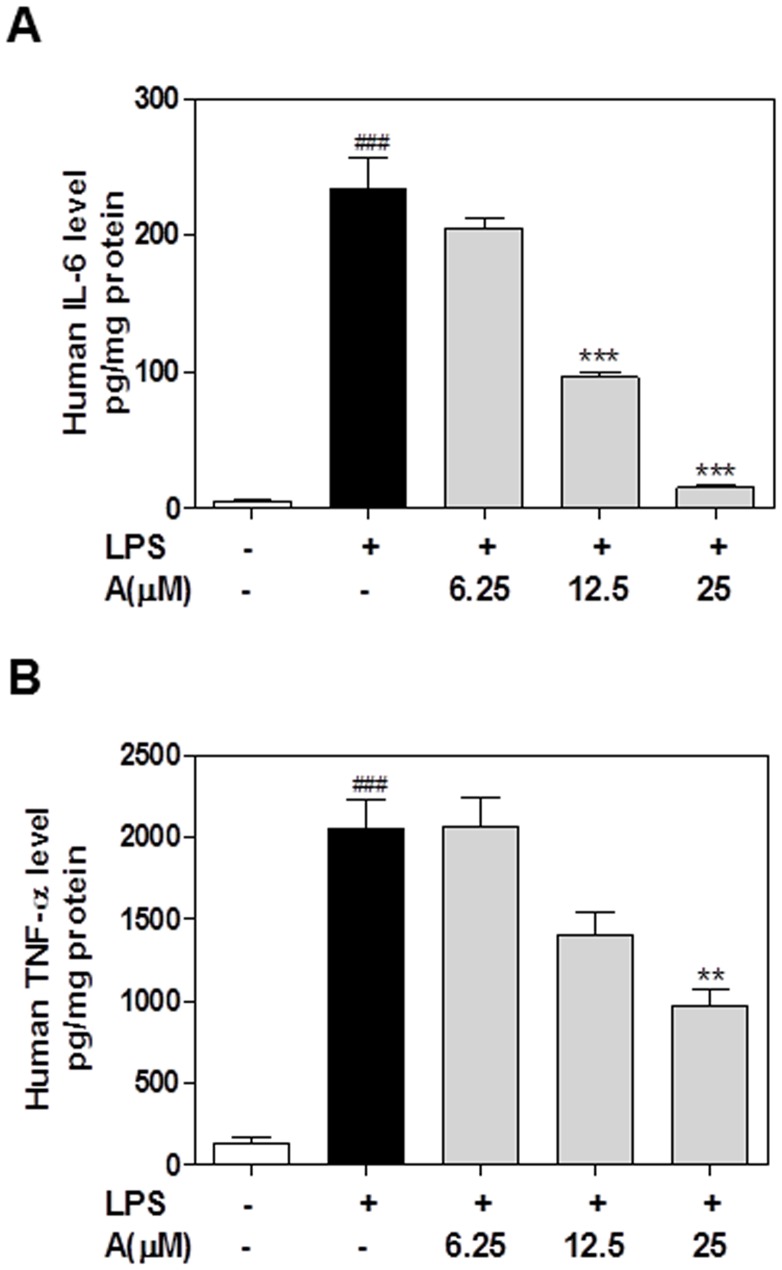
Effect of apigenin on LPS-induced IL-6 and TNF-α protein expression in human THP-1-derived macrophages. Cells were pretreated with different concentrations of apigenin (A, 6.25, 12.5,25 µM) for 2 h and then treated with LPS (100 ng/mL) for 24 h. At the end of treatment, each cell culture medium was collected. The protein levels of IL-6 and TNF-α were determined by ELISA as described under “Methods”. Values are mean ± S.E. of three independent experiments. Statistical significance relative to vehicle control, ###p<0.001; Statistical significance relative to LPS group, **p<0.01, ***p<0.001. **A**. IL-6; **B**.TNF-α.

**Figure 7:**
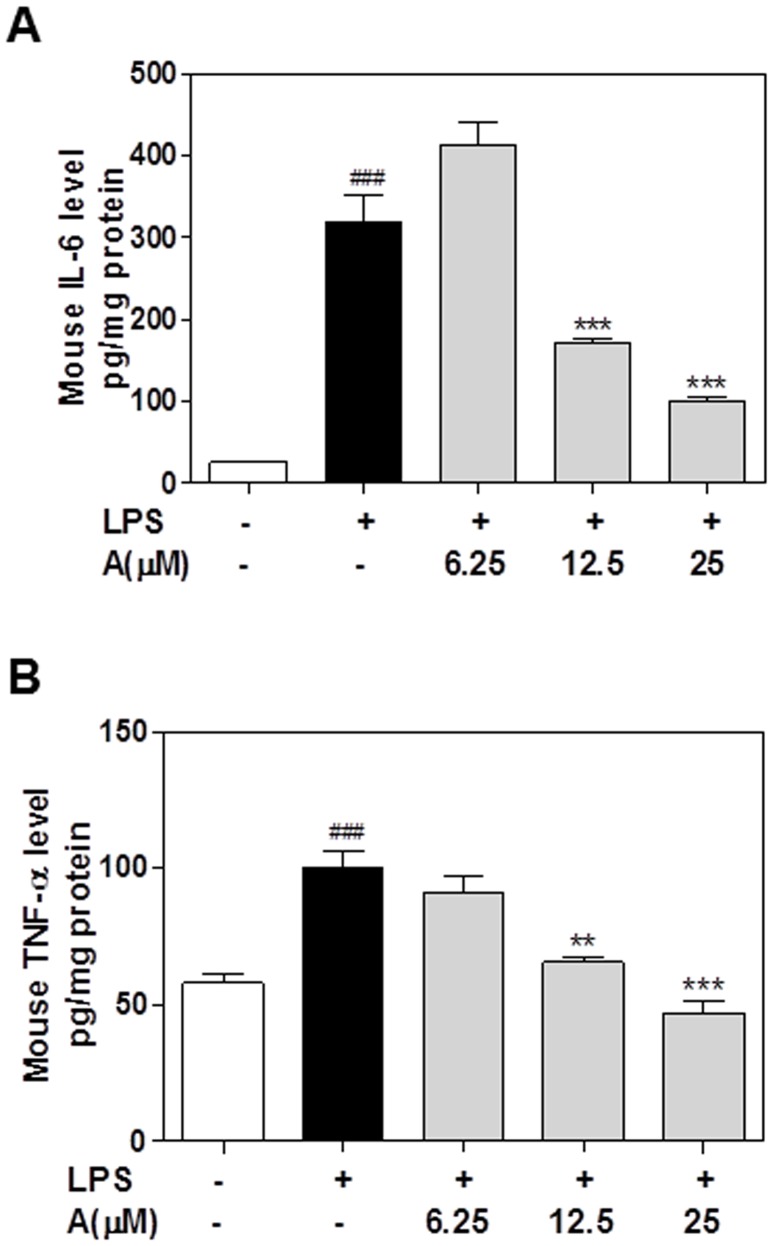
Effect of apigenin on LPS-induced IL-6 and TNF-α protein expression in in mouse J774A.1 macrophages. Cells were pretreated with different concentrations of apigenin (A, 6.25, 12.5,25 µM) for 2 h and then treated with LPS (100 ng/mL) for 24 h. At the end of treatment, each cell culture medium was collected. The protein levels of IL-6 and TNF-α were determined by ELISA as described under “Methods”. Values are mean ± S.E. of three independent experiments. Statistical significance relative to vehicle control, ###p<0.001; Statistical significance relative to LPS group, **p<0.01, ***p<0.001. **A**. IL-6; **B**.TNF-α.

In order to determine whether the inhibition of the mRNA expression of pro-inflammatory cytokines by apigenin is correlated to the reduction of protein levels, we measured the TNF-α and IL-6 secreted into cell culture media using ELISA. As shown in Fig.6, apigenin significantly reduced LPS-induced secretion of IL-6 in human THP-1-derived macrophages. However, apigenin was less potent in regulating TNFα expression. Similar results were obtained using mouse J774A.1 macrophages (Fig.7).

**Figure 8:**
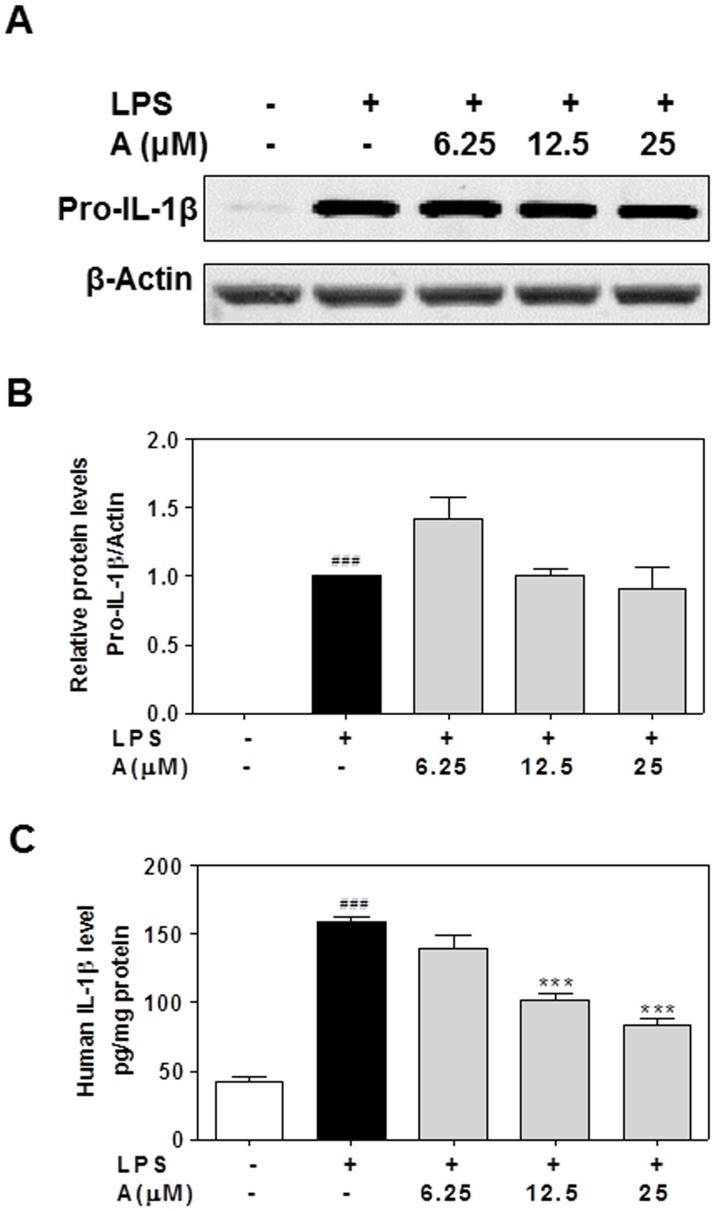
Effect of apigenin on LPS-induced IL-1β protein expression and maturation in human THP-1-derived macrophages. Cells were pretreated with apigenin (A, 6.25, 12.5, 25 µM) for 2 h and then treated with LPS (100 ng/mL) for 24 h. The Pro-IL-1β protein level was detected by Western blot analysis. β-Actin was used as a loading control. The mature IL-1β protein level was detected by ELISA as described under “Methods”. Values are mean ± S.E. of three independent experiments. Statistical significance relative to vehicle control, ###p<0.001; Statistical significance relative to LPS group, ***p<0.001. **A**. Representative immunoblots of Pro-IL-1β and β-Actin; **B**. The relative protein levels of pro-IL-1β were analyzed using Odyssey V3.0 software. **C**. Mature IL-1β level in the media.

The mature IL-1β production is rigorously controlled by expression, maturation and secretion. The pro-inflammatory stimuli induces expression of the inactive IL-1β precursor (pro-IL-1β), which lacks a classic signal peptide and is further processed into mature active IL-β by an intracellular cysteine protease, caspase-1, and secreted from the cell [28], [29]. To determine whether apigenin had any effect on pro-IL-1β protein expression and IL-1β maturation, we measured the intracellular pro-IL-1β protein levels by Western blot analysis and the secreted mature IL-1β protein levels in culture media by ELISA. As shown in Fig.8A and B, LPS significantly increased pro-IL-1β protein levels, but apigenin had no inhibitory effect on pro-IL-1β protein expression. However, the LPS-induced secretion of mature IL-1β was dose-dependently inhibited by apigenin in human THP-1-derived macrophages (Fig.8C). These results suggest that apigenin may regulate the maturation of IL-1β by targeting intracellular caspase-1.

### Effect of apigenin on caspase-1 activation in LPS-treated macrophages

**Figure 9:**
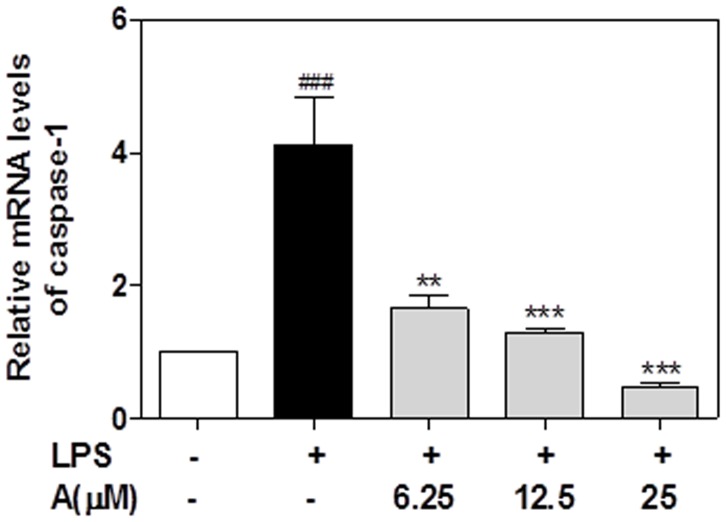
Effect of apigenin on LPS-induced caspase-1 mRNA expression in mouse J774A.1 macrophages. Cells were pretreated with different concentrations of apigenin (A, 6.25, 12.5,25 µM) for 2 h and then treated with LPS (100 ng/mL) for 24 hours. Total cellular RNA was isolated and reverse transcribed. The relative mRNA level of caspase-1 was detected by real-time RT-PCR as described under “Methods”. Values are mean ± S.E. of three independent experiments. Statistical significance relative to vehicle control, ##p<0.01; ###p<0.001; Statistical significance relative to LPS group, **p<0.01, ***p<0.001.

**Figure 10:**
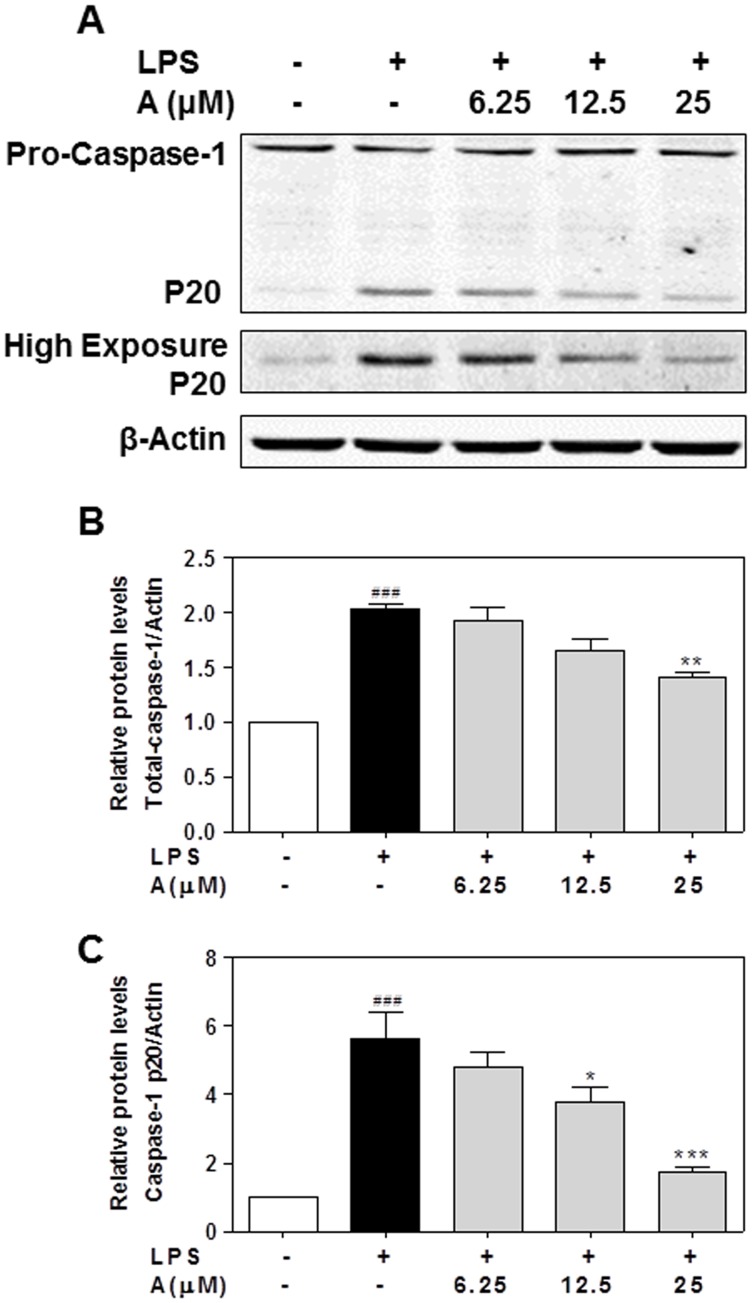
Effect of apigenin on caspase-1 activation in human THP-1-derived macrophages. Cells were pretreated with apigenin (A, 6.25, 12.5, 25 µM) for 2 h and then treated with LPS (100 ng/mL) for 24 h. Total cell lysates were prepared for Western blot analysis for caspase-1 protein level as described under “Methods”. β-Actin was used as a loading control. The relative protein levels of caspase-1 were normalized to β-Actin and analyzed using Odyssey V3.0 software. Values are mean ± S.E. of three independent experiments. Statistical significance relative to vehicle control, ###p<0.001; Statistical significance relative to LPS group, *p<0.05, **p<0.01, ***p<0.001. **A**. Representative immunoblots of Pro-caspase-1 and active caspase-1 p20. **B**. The relative protein levels of total caspase-1. **C**. The relative protein levels of active caspase-1.

Caspase-1 belongs to a family of nine cysteine proteases and is involved in regulating the inflammatory response by cleaving the precursors of several cytokines including IL-1β and IL-18. The activation of caspase-1 is the rate-limiting step in IL-1β-mediated inflammatory response. The activation of pro-caspase-1 results in the generation of the active tetrameric caspase-1 p20 and p10 fragments [28], [30]-[32]. In order to determine if apigenin exerts its effect on IL-1β maturation through regulating caspase-1 expression or activation, we first determined the effect of apigenin on LPS-induced mRNA expression of caspase-1. As shown in Fig. 9, LPS significantly increased the mRNA levels of caspase-1, which was completed inhibited by apigenin. The Western blot results further indicated that LPS not only increased the total caspase-1 protein expression, but also increased the processing of pro-caspase-1 into the active form (Fig. 10). However, apigenin had less effect on total caspase-1 protein levels. Consistent with the effect of apigenin on LPS-induced IL-1β maturation, apigenin significantly reduced LPS-induced activation of caspase-1 at the concentrations of 12.5 and 25 µM.

### Effect of apigenin on LPS-induced activation of inflammasome in macrophages

**Figure 11:**
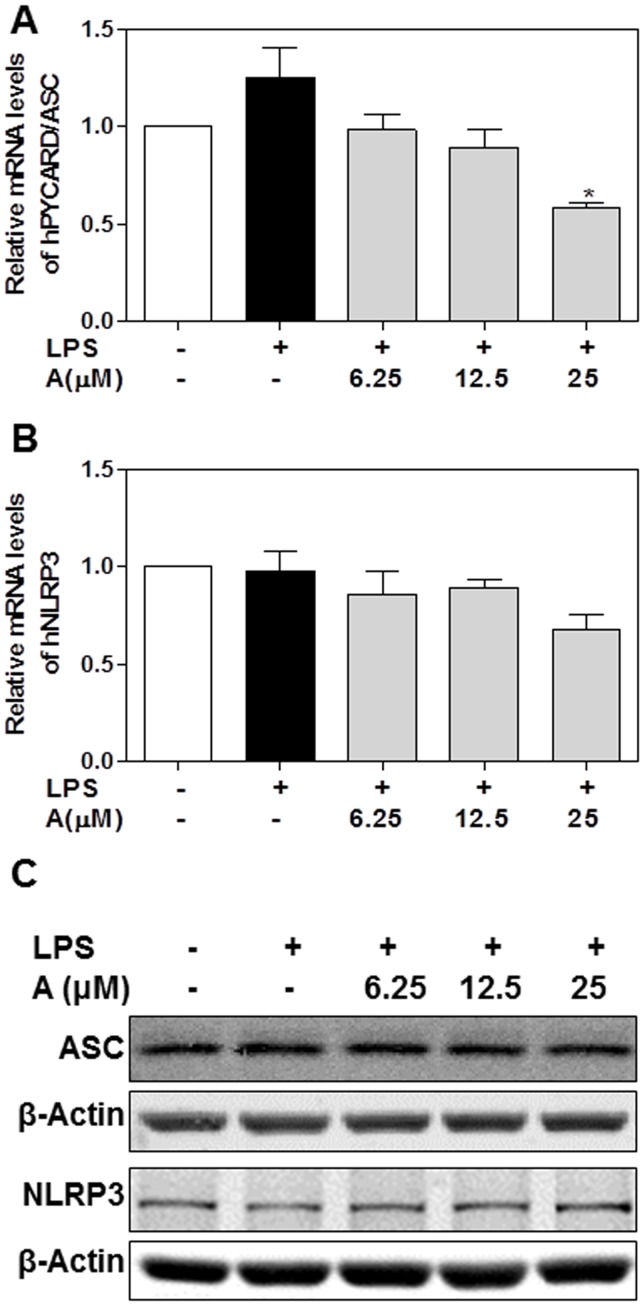
Effect of apigenin on the mRNA and protein expression of ASC and NLRP3 in human THP-1-derived macrophages. Cells were pretreated with apigenin (A, 6.25, 12.5, 25 µM) for 2 h and then treated with LPS (100 ng/mL) for 24 h. Total cellular RNA was isolated and reverse transcribed. The relative mRNA levels of PYCARD/ASC and NLRP3 were detected by real-time RT-PCR, as described under “METHODS”. Values are mean ± S.E. of three independent experiments. Statistical significance relative to LPS group, #p<0.05. **A**. The mRNA level of PYCARD/ASC; **B**. The mRNA levels of NLRP3. **C**. The protein levels of ASC and NLRP3 were detected by Western blot analysis as described under “EXPERIMENTAL PROCEDURES”. The representative images of ASC and NLRP3 are shown.

**Figure 12:**
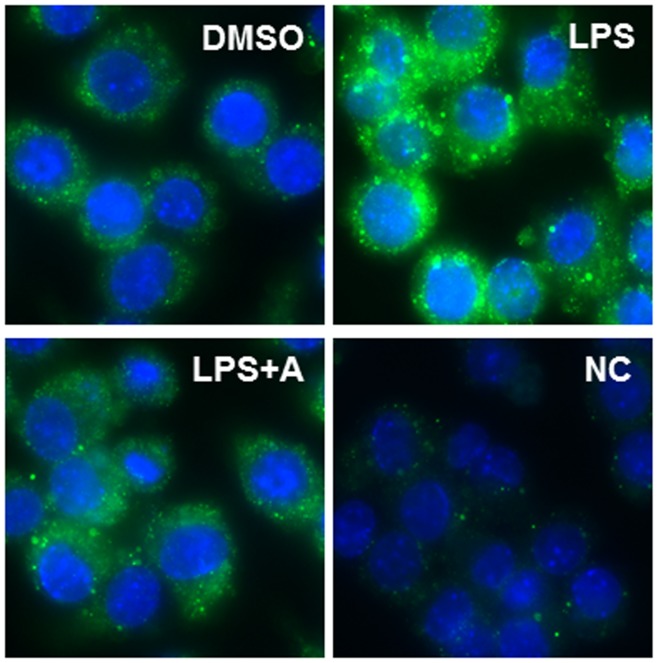
Effect of apigenin on LPS-induced oligomerization of ASC in human THP-1-derived macrophages. Cells were pretreated with apigenin (A, 25 µM) for 2 h and then treated with LPS (100 ng/mL) for 24 h. Cells were fixed and analyzed for ASC expression using immunofluorescence staining, as described under “METHODS”. The representative images for each treatment are shown.

The production of mature IL-1β depends on the formation of NLRP3 inflammasome, which is a multi-protein complex and comprised of a NLRP3 sensor, an ASC/PYCARD adaptor and caspase-1 [33]. LPS-induced activation of pro-caspase-1 requires the formation of a functional NLRP3 inflammasome [34], [35]. In order to determine whether apigenin had any effect on the NLRP3 inflammasome, we measured the mRNA and protein levels of NLRP3 and ASC. As shown in Fig.11, LPS had no significant effect on NLRP3 and ASC/PYCARD expression. While apigenin reduced the mRNA level of ASC/PYCARD at the 25 µM concentration, the protein levels of ASC and NLRP3 remained unchanged. To further elucidate the potential mechanism by which apigenin inhibits LPS-induced maturation of IL-1β, we examined the effect of apigenin on the assembly of the NLRP3 inflammasome. The human THP-1-derived macrophages were pretreated with apigenin (25 µM) for 2 h, and then treated with LPS (100 ng/ml) for 24 h. The formation of ASC specks was monitored using immunofluorescence staining with a specific antibody against ASC. As shown in Fig.12, LPS significantly induced the formation of ASC specks; however, pretreatment of the cells with apigenin significantly prevented the LPS-induced increase in the formation of ASC specks. These results suggest that apigenin inhibits the oligomerization of ASC and thus, interferes with the assembly of a functional inflammasome.

### Effect of apigenin on stability of IL-6 mRNA in LPS-treated macrophages

**Figure 13:**
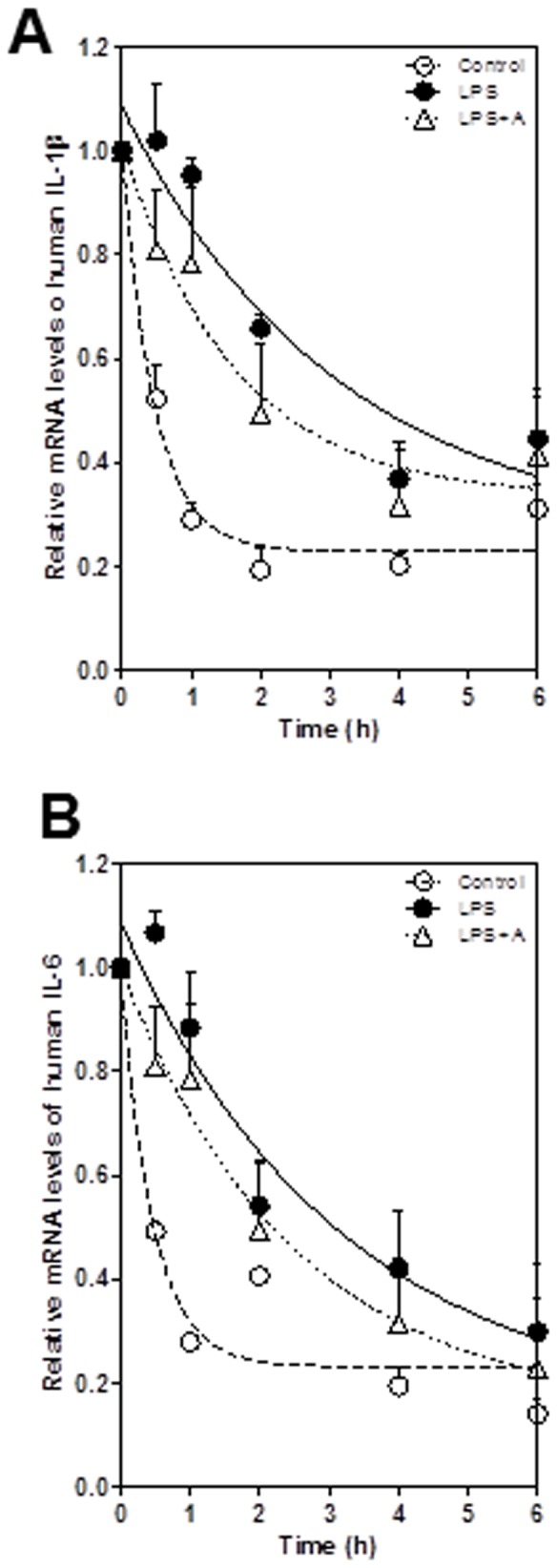
Effects of apigenin on LPS-mediated mRNA stabilization in human THP-1-derived macrophages. Cells were pretreated with apigenin (A, 25 µM) for 2 h and then treated with LPS (100 ng/mL) for 2 h, followed by treatment with actinomycin D (5 µg/ml). Total cellular RNA was isolated at 0, 0.5,1, 2, 4 and 6 h after actinomycin D treatment. The mRNA levels of IL-1β and IL-6 were determined by real-time RT-PCR as described under “METHODS”. Values are the means ± S.E. from three independent experiments. **A**. IL-1β; **B**. IL-6.

**Figure 14:**
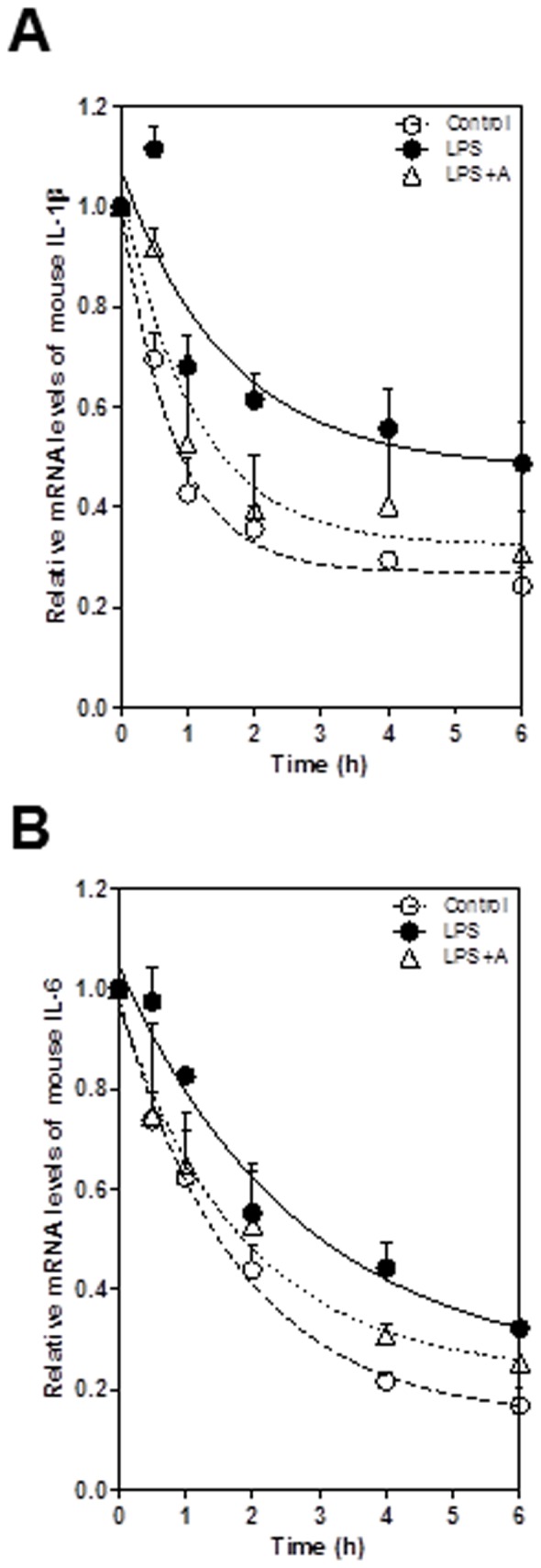
Effects of apigenin on LPS-mediated mRNA stabilization in mouse J774A.1 macrophages. Cells were pretreated with apigenin (A, 25 µM) for 2 h and then treated with LPS (100 ng/mL) for 2 h, followed by treatment with actinomycin D (5 µg/ml). Total cellular RNA was isolated at 0, 0.5,1, 2, 4 and 6 h after actinomycin D treatment. The mRNA levels of IL-1β and IL-6 were determined by real-time RT-PCR as described under “METHODS”. Values are the means ± S.E. from three independent experiments. **A**. IL-1β; **B**. IL-6.

Post-transcriptional regulation is a major control point for the expression of many inflammatory cytokines with short half-lives including IL-6 and IL-1β [36], [37]. Our previous studies have shown that Berberine inhibits HIV protease inhibitor-induced IL-6 expression through regulating its mRNA stability in macrophages [23]. In order to identify the other potential mechanisms by which apigenin inhibits LPS-induced IL-6 and IL-1β expression in macrophages, we examined the effect of apigenin on stability of IL-6 and IL-1β mRNA in LPS-stimulated human THP-1-derived macrophages. As shown in Fig.13, apigenin significantly inhibited LPS-induced increase of IL-6 and IL-1β mRNA stability. Similar results were obtained in mouse J774A.1 macrophages (Fig.14).

### Effect of apigenin on ERK1/2 phosphorylation and NF-κB activation

**Figure 15:**
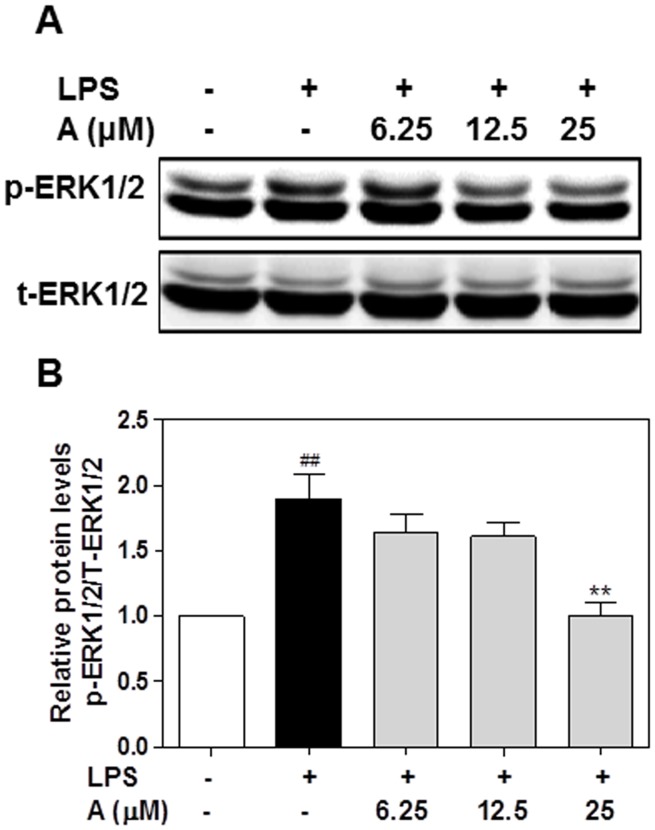
Effect of apigenin on ERK1/2 activation in human THP-1-derived macrophages. Cells were pretreated with apigenin (A, 6.25, 12.5, 25 µM) for 2 h and then treated with LPS (100 ng/mL) for 24 h. Total cell lysates were prepared for Western blot analysis for phospho(p)-ERK and total (T)-ERK as described under “METHODS”. The density of the immunoblots was analyzed using Odyssey V3.0 software and normalized to T-ERK. **A**. Representative images of immunoblots of p-ERK1/2 and T-ERK1/2; **B**. Relative protein levels of p-ERK.

MAPK-mediated signaling pathways play critical roles in LPS-induced proinflammatory cytokine production [38]. In macrophages, LPS activates ERK1/2, p38 MAPK, and Jun N-terminal kinase (JNK) [38]. Activation of ERK1/2 has been shown to promote LPS-induced IL-6 production [39]. Our previous studies also showed that ERK activation is responsible for HIV protease inhibitor-induced IL-6 expression in macrophages [22]. It also has been reported that several flavonoids inhibit LPS-induced inflammatory response through inhibiting ERK1/2 activation [40]–[44]. As shown in Fig.15, LPS-induced ERK activation was inhibited by apigenin in humanTHP-1-derived macrophages.

**Figure 16:**
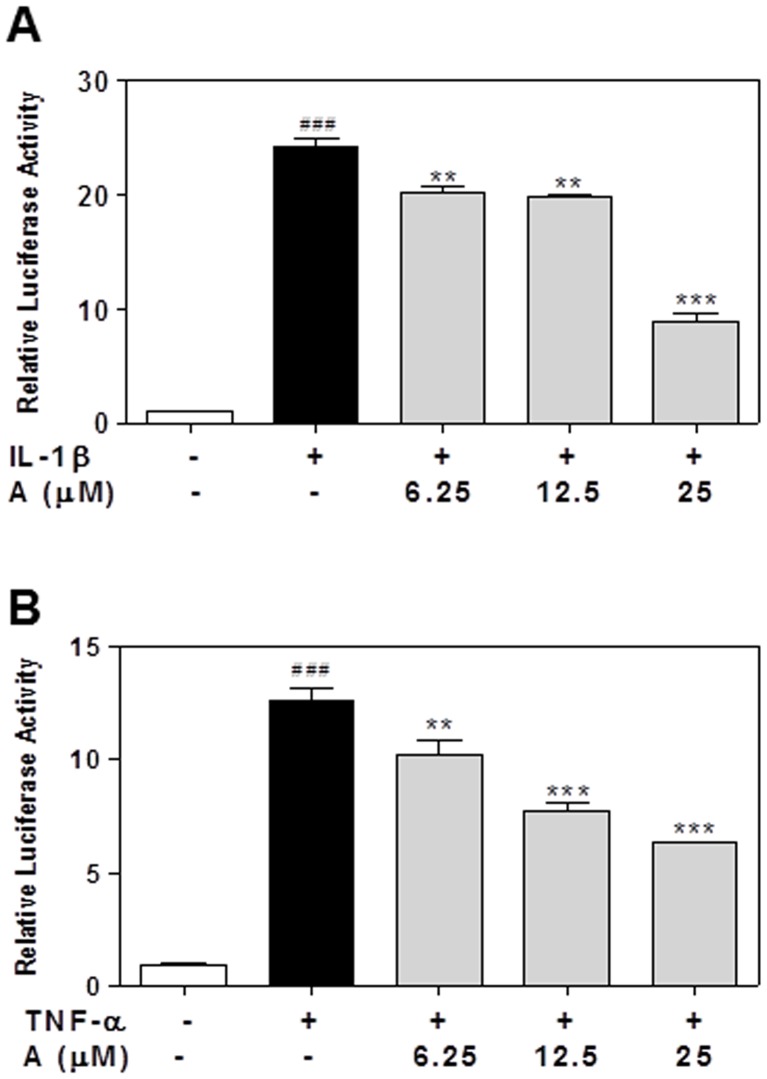
Effect of apigenin on pro-inflammatory cytokine-induced activation of NF-κB. Human 293 Cells were stably transfected with pGL4.32 [luc2P/NF-κB-RE/Hygro] luciferase reporter. Cells were pretreated with apigenin (6.25, 12.5,25 µM) for 2 h and then treated with human IL-1β (10 ng/mL) or TNF-α (10 ng/mL) for 4 h. The luciferase activity was detected as described under “METHODS”. Values are mean ± S.E. of three independent experiments. Statistical significance relative to vehicle control, ###p<0.001; Statistical significance relative to LPS group, **p<0.01, ***p<0.001. **A**. IL-1β; **B**. TNF-α.

Another control point of proinflammatory gene expression is the NF-κB activation [45]. Inhibition of NF-κB activation represents an important mechanism by which flavonoids inhibit LPS-induced production of proinflammatory cytokines such as TNF-α, IL-6 and IL-1β [44]. In order to determine whether NF-κB is also the target of apigenin, HEK293 cells were stably transfected with a luciferase reporter, which contains five copies of an NF-κB response element. As shown in Fig.16, both IL-1β and TNF-α significantly activated the NF-κB, which was inhibited by apigenin in a dose-dependent manner. In the cells stably transfected with the luciferase control vector, IL-1β and TNF-α had no effect on luciferase activity (Data not shown).

## Discussion

The principle findings of this study elucidated the important cellular mechanisms underlying the anti-inflammation effect of apigenin, a natural flavonoid. Apigenin inhibits LPS-induced inflammatory response by i) modulating inflammasome assembly; ii) reducing the mRNA stability; iii) inhibiting ERK1/2 and NF-κB activation in macrophages.

Macrophages are the most important immune cells involved in the inflammatory response. Macrophages can be activated by many invaders, such as bacteria and virus, and activated macrophages can produce numerous proinflammatory cytokines. Although the acute inflammatory response helps restore cellular homeostasis, prolonged activation of the inflammatory response will result in severe tissue injury and organ failure [46]. Chronic inflammation is an important risk factor for various human diseases. Targeting reduction of chronic inflammation is an effective strategy to prevent pathological progression of chronic diseases. Flavonoids are the most commonly found polyphenol compounds in fruits and vegetables. Epidemiological studies have shown that high intake of diets rich in flavonoids can prevent many chronic diseases including cardiovascular disease, metabolic disease, allergy, and cancer [9], [47]–[50]. A variety of mechanisms have been proposed by which flavonoids prevent and attenuate inflammatory responses and serve as possible cardioprotective, neuroprotective and chemopreventive agents [8], [9], [11], [47], [48], [51]–[53]. Apigenin has been reported as an important dietary flavonoid with strong chemopreventive and anti-inflammatory activities [13]–[19]. Previous studies reported that apigenin significantly decreased TNF-α, IL-6, and IL-1β mRNA levels in LPS-activated mouse J774.2 macrophages [54]. However, the cellular/molecular mechanisms by which apigenin inhibits the inflammatory response remain to be fully identified.

In the present study, we were able to identify the major target genes regulated by apigenin in the LPS-induced inflammatory response in macrophages by using the newly developed PrimePCR array. The results indicated that apigenin not only inhibited LPS-induced increase of the major pro-inflammatory cytokines IL-1β and IL-6, chemokine CCL-5 and two adhesion molecules ICAM-1 and VCAM-1, but also prevented LPS-induced decrease of anti-inflammatory cytokine IL-10 (Fig.2–5). The cytotoxicity study indicated that Apigenin had no toxic effect on macrophages at a dose of 25 µM (Data not shown). By using both human and mouse macrophages, we were able to identify the important mechanisms underlying apigenin's anti-inflammatory activities. As shown in Figs. 4–7, we first demonstrated that apigenin specifically targets ASC and interferes with the NLRP3 inflammasome formation and subsequently inhibits caspase-1 activation in macrophages. NLRP3 inflammasome-mediated production of mature IL-1β has been recently identified as a critical mediator in the disease progression of a variety of metabolic diseases [55]. IL-1β functions as a master cytokine that can further induce the expression of other pro-inflammatory cytokines, such as IL-6 and TNF-α, chemokines, adhesion molecules and other inflammation-associated molecules to amplify the inflammatory response [29]. Therefore, the NLRP3 inflammasome represents an important therapeutic target for inflammatory diseases.

The expression of pro-inflammatory cytokines is regulated at multiple levels, including post-transcriptional regulation by modulating mRNA stability. It has been reported that chloroquine reduced the mRNA levels of IL-1β and IL-6 mRNA by decreasing their stability in LPS-stimulated human monocytes/macrophages [56]. In the present study, we also identified that post-transcriptional regulation of mRNA stability also contributes to apigenin's anti-inflammatory activities.

Numerous studies have shown that both MAPKs and NF-κB signaling pathways are involved in activation of an inflammatory response [57], [58]. Thus, inhibition of the LPS-stimulated signal transduction cascades has been proposed as a promising target for the treatment of inflammation. It has been reported that apigenin inhibits LPS-induced inflammation through inhibition of NF-κB activation by hypophosphorylation of Ser536 in the p65 subunit in an *in vivo* mouse model [19]. Consistent with previous findings, we also found that apigenin significantly inhibited LPS-induced ERK1/2 and NF-κB activation in human THP-1 macrophages.

According to the previous research of the structure–activity relationship of flavonoids and their anti-inflammatory effects, the C2–C3 double-bond along with 4-oxo functional group of the C-ring is essential to the higher anti-inflammatory effect. In addition, the hydroxylations at positions 5, 7, 3′, 4′ are very important for strong anti-inflammatory effects [59]. Based on the above principles, apigenin has promising anti-inflammatory structure (Fig.1) based on the above principles and its anti-inflammatory effect is further verified in the current study.

Natural plants are the endless sources of medicines, which play an essential role in healthcare. The documentation of using plant-based medicine in health protection and disease control dates back to thousands of years ago [60]. Recent advances in understanding of the pathologies of various human diseases have shifted the drug discovery paradigm from “one-disease-one-drug” to a “combinational strategy”, which opens up a unique opportunity for traditional plant medicine [61]–[63]. During the last decade, the pharmaceutical companies face shrinking pipelines of new drug candidates and increasing failure of chemically synthesized drugs, due to low efficacy and severe side effects. The focus of new drug discovery efforts is shifting from the laboratory bench back to nature. Plant medicine has been and will continue to be a rich resource in the development of new drugs [64]. However, the major obstacles for the advancement of plant medicine in new drug development are a lack of scientific evidence of functional mechanisms, unknown toxicity and uncertain drug-drug interactions. Our findings in the present study provides important scientific evidence for the potential application of apigenin as a therapeutic agent for inflammatory diseases.
